# Dental Calculus Is Associated with Death from Heart Infarction

**DOI:** 10.1155/2014/569675

**Published:** 2014-01-09

**Authors:** Birgitta Söder, Jukka H. Meurman, Per-Östen Söder

**Affiliations:** ^1^Department of Dental Medicine, Karolinska Institutet, P.O. Box 4064, 14104 Huddinge, Sweden; ^2^Institute of Dentistry and Department of Oral and Maxillofacial Diseases, University of Helsinki and Helsinki University Central Hospital, PB 41, 00014 Helsinki, Finland

## Abstract

*Objectives*. We studied whether the amount of dental calculus is associated with death from heart infarction in the dental infection—atherosclerosis paradigm. *Materials*. Participants were 1676 healthy young Swedes followed up from 1985 to 2011. At the beginning of the study all subjects underwent oral clinical examination including dental calculus registration scored with calculus index (CI). Outcome measure was cause of death classified according to WHO International Classification of Diseases. Unpaired *t*-test, Chi-square tests, and multiple logistic regressions were used. *Results*. Of the 1676 participants, 2.8% had died during follow-up. Women died at a mean age of 61.5 years and men at 61.7 years. The difference in the CI index score between the survivors versus deceased patients was significant by the year 2009 (*P* < 0.01). In multiple regression analysis of the relationship between death from heart infarction as a dependent variable and CI as independent variable with controlling for age, gender, dental visits, dental plaque, periodontal pockets, education, income, socioeconomic status, and pack-years of smoking, CI score appeared to be associated with 2.3 times the odds ratio for cardiac death. *Conclusions*. The results confirmed our study hypothesis by showing that dental calculus indeed associated statistically with cardiac death due to infarction.

## 1. Introduction 

The dentogingival region is the natural habitat for oral microorganisms which form biofilms (dental plaque) on tooth surfaces. These microorganisms play a role in the etiology of dental diseases. They also link to general health and cause systemic diseases due to direct or hematogenic spread of bacteria and their toxins, with subsequent upregulation of cytokines and inflammatory mediators [1]. Our group has earlier shown that periodontal disease associated with the development of early atherosclerotic carotid lesions [2].

The average microscopic count of bacteria in unmineralized dental plaque has been calculated to be up to 2.1 × 10^8^/mg wet weight [3, 4]. Oral microbiota contains hundreds of species [5]. A dental biofilm is formed on the nonshedding oral surfaces, namely, on teeth, dental restorations, and prostheses. The biofilm may absorb calcium or phosphate ions from saliva and gingival crevicular fluid resulting in dental calculus [6]. Hence, dental calculus is primarily composed of calcium phosphate mineral salts covered by unmineralized bacterial biofilm. Although the first evidence of calcification in the biofilm is seen after only a few days, mature calculus requires months or even years to develop [7, 8]. Lactate dehydrogenase and alkaline and acid phosphatase activities have been detected in dental plaque suggesting an enhanced calcification by the plaque enzymes [9]. Viable aerobic and anaerobic bacteria have been detected in supragingival calculus while subgingival calculus provides an excellent environment for further microbial adhesion and growth [10]. Periopathogens such as *Aggregatibacter actinomycetemcomitans*, *Porphyromonas gingivalis*, and *Treponema denticola *have been found within the deep recesses of the structural channels and lacunae of both supra- and subgingival calculus [11, 12]. Bacteria are not essential for calculus formation, but they facilitate its development [13]. Hence, high amount of calculus indicates that oral hygiene has been poor for months or even years [14]. Subsequently, a high dental calculus index score might indicate a long-lasting oral infection burden which causes upregulation of systemic inflammatory reactions. These, in turn, may be involved in the development of atherosclerosis and finally lead to death from heart infarction.

Acute myocardial infarction with subsequent left ventricular dysfunction and heart failure indeed continues to be the major cause of morbidity and mortality in industrialized countries [15]. Cardiovascular diseases constitute the greatest major health problem also in Sweden where mortality from coronary disease is high [16]. In 1987–2010, the number of registered cases with incident episodes or attacks was 946 000. In the same period, 715 000 persons had verified acute myocardial infarction resulting in death of 341 000 subjects [17]. There also seems to be an association between periodontal disease and heart infarction [18, 19]. Our group has previously shown that young individuals with periodontitis and missing molars, which indicate a history of chronic dental infections, have an increased risk for premature death from diseases of the circulatory system [2].

In the present study we hypothesized that dental calculus is associated with premature death from heart infarction. The specific aim was to investigate in our 26-year prospective study whether the amount of dental calculus is associated with cardiac death. The unique national population and hospital and death registers of Sweden made conducting this study possible.

## 2. Material and Methods

### 2.1. Study Population and Oral Health Parameters

**Figure 1:**
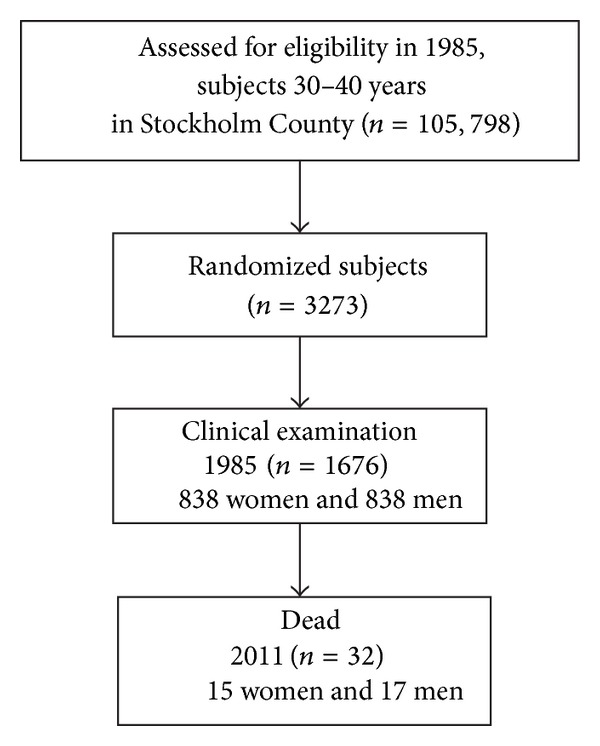
Study profile.

In 1985, we undertook a study comprising a random sample of 3 273 persons aged 30–40 years. The subjects were selected from the registry file of all inhabitants of Stockholm County born on the 20th of any month from 1945 to 1954 (*n* = 105 798). The registry file including all individuals born on the 20th of any month, from 1985 and ongoing, is a unique population register in Sweden. They were informed about the purpose of the study and offered a clinical oral examination. In total, 1 676 individuals (51.2%), 838 women and 838 men, underwent a clinical oral examination and answered a questionnaire including information on dental visits and smoking. These 1 676 subjects were then included in the present study. The study profile is given in Figure 1. The following oral health parameters were recorded: number of remaining teeth excluding third molars, gingival inflammation around every tooth using the gingival index (GI) [20], oral hygiene status using the plaque index (PLI) [21], and assessment of all six surfaces of six representative teeth using the calculus index (CI) [22]. PLI, CI, and GI are all measured on an ordinal scale, with the units 0, 1, 2, and 3. Gingival crevices were measured using a periodontal probe and recorded to the nearest higher millimetre for six sites of each tooth. All subjects answered a questionnaire concerning topics such as regular dental visits and use of tobacco. Smoking was expressed in the analyses as pack-years of smoking.

### 2.2. Socioeconomic and Mortality Data

The cumulative causes of death of the 1 676 subjects followed up from 1985 to 2011 were obtained from the Centre of Epidemiology, Swedish National Board of Health and Welfare, Sweden. The data regarding the causes of death were classified according to the WHO International Statistical Classification of Diseases and Related Health Problems (ICD-9 and ICD-10). Socioeconomic data were obtained from the National Statistics Centre, Örebro, Sweden. The data regarding the causes of death as well as socioeconomic status were obtained from the registry files including data of persons born on the 20th of any month from 1985 and ongoing. This kind of register is uncommon in other countries and indeed unique for Sweden.

### 2.3. Statistical Analysis

Unpaired *t*-test, Chi-square tests, median and interquartile range (IQR) statistics, multiple regression analysis, and multiple logistic regression analysis were applied when appropriate. We used multiple logistic regression analysis to compare the incidence of mortality according to the state of oral health at baseline, while simultaneously controlling for several potential confounding variables. We included in the model the variables of age, sex, education, income, socioeconomic status, smoking (pack-years of smoking), dental visits, dental plaque, gingival inflammation, dental calculus, pocket depth, and hospitalization. The outcome variable was death from heart infarction. Differences between data sets with a probability of <0.05 were regarded as significant. All *P* values are two tailed, and confidence intervals (CIs) were calculated at the 95% level. All statistical analyses were performed using the PASW Statistics software package, version 20 (PASW Inc., Chicago, Illinois, USA).

### 2.4. Ethics Approval

Ethics approval was provided by the Ethics Committee of the Karolinska Institutet, and Huddinge University Hospital, Sweden, had approved the study protocol (permit 2007/1669-31). The study is in accordance with the Declaration of Helsinki as revised in 1983.

## 3. Results

By 2011, of the 1 676 subjects originally examined, 32 had died from heart infarction; of them 15 (46.8%) were women and 17 (53.2%) were men. The difference between genders was not significant (*P* = 0.55). For comparison, of the total cohort of 3 273 subjects 240 (7.3%) had died, 158 men at the mean age of 51.7 (±7.7 SD) years and 82 women at the mean age of 53.1 (±6.6 SD) years, respectively, (*P* = 0.19). In the present study cohort women died at a mean age of 61.5 (±2.6 SD) years and men at 61.7 (±2.6 SD) years, respectively (*P* = 0.35). Of these, 19 men and 7 women, respectively, died from their first heart infarction, three men from their second infarction, two men from their third infarction, and one man from his seventh infarction. The specific causes of cardiac deaths are given in Table 1.

Demographic data and risk indicators at the baseline examination of the 1 676 subjects in 1985 are presented in Table 2. Survivors were characterized by higher age while the deceased patients were characterized by greater CI scores. In addition, when the data were analysed using median and IQR statistics for CI scores and missing teeth values, CI score of the deceased was 0.42 and missing teeth value 1.09, respectively, while those of the survivors were CI 0.17 and missing teeth 0.67, respectively (*P* = 0.083). Median of missing teeth among the deceased was 0 with IQR 2, while respective values of the surviving were 1 and 2 (*P* value not computed). We also registered the CI scores of the 1 676 subjects who either had died or were alive in the follow-up examination years of 1996, 1998, 2000, 2001, and 2009. The results are given in means in Table 3. Those who were alive in 2009 had had almost the same CI score during the whole observation period; namely, in 1996 their CI score was 0.46 compared with CI score 0.45 in 2009, respectively, whereas the deceased subjects showed significantly higher scores: in 1996 their CI score was 0.61 compared with CI score 0.67 in the year 2009 examination, respectively (*P* < 0.01).

In the multiple logistic regression analysis, CI score appeared to be the only principal independent predictor for cardiac death during the follow-up. The results are given in Table 4. The high CI score was associated with 2.30 times the odds of premature death from heart infarction (*P* = 0.038). The CI score in the model is based on each unit increase in the index. Other factors considered in the model exerted no significant independent influence on this outcome variable.

## 4. Discussion

This study addressed for the first time dental calculus as a risk marker for mortality from heart infarction by evaluating the relationship between the clinically recorded CI scores and cardiac deaths 26 years after the baseline clinical oral examination. Our results confirmed the study hypothesis by showing that dental calculus indeed associated with cardiac death. The role of infections in heart disease in general has been known for a long time, [23] and the putative mechanisms involved have been thoroughly discussed [24]. Lockhart et al. [25] recently summarized the role of periodontal disease in atherosclerosis, describing an independent but not causal association between these two diseases. Based on our results, high amount of dental calculus over time may play a role in premature death in heart infarction. Removal of dental calculus at regular intervals is therefore important in reducing the burden of bacteremia and endotoxemia of oral origin.

Nevertheless, some comments should be made concerning the reliability of the results. Our subjects were randomly chosen at baseline to avoid selection bias. The subject pool was representative of the ethnically homogenous Swedish adult population, with an age range of 10 years to limit the influence of age differences. The study was of longitudinal prospective design, with a group of subjects with oral health status documented 26 years earlier. A weakness is the lack of data of certain atherosclerosis risk factors such as body mass index and serum lipid values which due to the nature of the study were not available for analyses.

As said, cardiovascular diseases continue to constitute a major health problem in Sweden, [16] even though the risk for coronary heart disease, mainly myocardial infarction, has decreased by about 20% during the period of 1987–1997. The decline in cardiovascular mortality is the main explanation for the increased life expectancy in recent years in Sweden which in 2010 was 83.6 years for women and 79 years for men [26]. However, if the Swedish results are compared with respective international data there is still high mortality from coronary heart disease in this country being, for instance, twice as high as in France [16].

The deceased women in our study cohort were expected to live 22.1 years longer and the deceased men 17.3 years longer according to population demographics in Sweden [26]. Hence, the deaths recorded here could indeed be termed premature and the results further show a need for preventive measures; here maintaining good oral health might play an important role. It needs to be reemphasized that in the subjects who had been healthy in the 1985 examination and also had then no signs of oral diseases but who had died by the year 2011, a voluminous amount of calculus had been accumulating in their gingival crevices. Hence, a potential source of dentogenic infection had been present for years (Table 3). CI scores of the subjects who were alive in 2009 were significantly lower than scores of the patients who had died.

Lockhart et al. [25] showed in their infective endocarditis study on 195 patients that those with mean dental plaque and CI scores of 2 or greater were at 3.78- and 4.43-fold increased risk, respectively, of developing bacteremia. However, oral hygiene habits of most individuals seem insufficient to prevent calculus formation [27, 28]. In practice, dental calculus cannot be removed by tooth brushing or flossing but calls for the professional skills of a dentist or dental hygienist. In contrast to the epithelial surfaces of the mouth tooth surfaces are not self-cleaning, and if dental plaque is not mechanically removed calculus may develop [7, 8, 28].

In the present study statistical analysis was performed with adjustment for several demographic variables and established risk markers for mortality, including education, pack-years of smoking, frequency of dental visits, income level, socioeconomic status, gingival inflammation, and periodontal pocket depth. Therefore, none of these variables confounded the association observed between age, gender, amount of dental calculus, and cardiac death. Further studies are nevertheless needed to determine whether a causal element exists in the reported association. Based on the present findings new strategies for prevention and practical health recommendations might be warranted.

## 5. Conclusions 

The results confirmed our study hypothesis by showing that high dental calculus index scores associated statistically with premature death in heart infarction.

## Figures and Tables

**Table 1:** Cardiac diagnoses of the 32 subjects who had died from heart infarction by the year 2011.

Diagnosis	Men (*n*)	Women (*n*)
ICD-9		
410 Acute myocardial infarction (ICD-9, 410)	2	0
410.0 Acute myocardial infarction of anterolateral wall (ICD-9, 410.0)	5	0
410.1 Acute myocardial infarction of other anterior wall (ICD-9, 410.1)	2	0
410.2 Acute myocardial infarction of inferolateral wall (ICD-9, 410.2)	2	0
410.9 Acute myocardial infarction, NOS (ICD-9, 410.9)	1	0
ICD-10		
0 Acute transmural myocardial infarction of anterior wall (ICD-10, I21.0)	5	0
1 Acute transmural myocardial infarction of inferior wall (ICD-10, I21.1)	2	0
2 Acute transmural myocardial infarction of other sites (ICD-10, I21.2)	1	0
3 Acute transmural myocardial infarction of unspecified site (ICD-10, I21.3)	1	0
4 Acute endocardial myocardial infarction (ICD-10, I21.4)	14	5
9 Acute myocardial infarction, unspecified (ICD-10, I21.9)	5	2

**Table 2:** Demographic clinical oral health data at the baseline examination in 1985 of 1676 subjects who were dead or alive by the follow-up year of 2011.

	Dead (*n* = 32) number, mean ± SD	Alive (*n* = 1644) number, mean ± SD	*P* value*
Gender (female/male)	15/17	823/821	NS
Age in 1985 (years)	35.3 ± 2.9	35.7 ± 2.9	<0.05
Education (compulsory school/higher)	7/25	284/1360	NS
Smoking (pack-year)	2592.8 ± 3873.5	3394.4 ± 4151.4	NS
Income (Swedish Crowns ×1000)	157.4 ± 60.0	188.3 ± 10.1	NS
Plaque index	0.78 ± 0.48	0.71 ± 0.49	NS
Gingival inflammation	1.28 ± 0.50	1.28 ± 0.53	NS
Calculus index	0.71 ± 0.80	0.45 ± 0.58	0.015
Periodontal pockets (*n*)	0	0.90 ± 2.87	0.001
Missing teeth	1.34 ± 1.88	1.26 ± 2.39	NS
Missing molars	0.53 ± 1.03	0.53 ± 1.23	NS

Data are expressed as mean ± SD.

*Fisher's exact *t*-test or Student's *t*-test for unpaired samples as appropriate.

**Table 3:** Dental calculus indices of the 1676 subjects who survived or died from heart infarction during the years 1996–2009.

Year	Alive (*n*)	Dead (*n*)	Dental calculus index among the surviving	Dental calculus index among the deceased	*P* value
1996	1653	18	0.46 ± (0.58 SD)	0.61 ± (0.61 SD)	NS
1998	1647	24	0.46 ± (0.58 SD)	0.61 ± (0.76 SD)	NS
2000	1642	29	0.46 ± (0.58 SD)	0.64 ± (0.75 SD)	NS
2001	1631	40	0.46 ± (0.58 SD)	0.65 ± (0.73 SD)	<0.05
2009	1598	73	0.45 ± (0.57 SD)	0.67 ± (0.76 SD)	<0.01

Data expressed as mean and ± SD.

**Table 4:** Results of multiple logistic regression analysis of the relationship between death from heart infarction as a dependent variable and calculus index as independent variable with control for age, gender, dental visits, dental plaque, periodontal pockets, education, income, socioeconomic status, and pack-years of smoking.

Dependent variable	Explaining variable	Beta	Chi-square	*P* value	OR (95% CI)
Death from heart infarction	Dental calculus	0.834	4.323	0.038	2.30 (1.05–5.06)

Cox and Snell *R* ^2^, final solution could not be determined; Nagelkerke *R* ^2^ = 0.049.
